# Involvement of Receptor Tyrosine Kinase Tyro3 in Amyloidogenic APP Processing and β-Amyloid Deposition in Alzheimer's Disease Models

**DOI:** 10.1371/journal.pone.0039035

**Published:** 2012-06-11

**Authors:** Yan Zheng, Qi Wang, Bing Xiao, Qingjun Lu, Yizheng Wang, Xiaomin Wang

**Affiliations:** Department of Physiology, Capital Medical University, Key Laboratory for Neurodegenerative Disorders of the Ministry of Education, Beijing, People's Republic of China; Nathan Kline Institute and New York University School of Medicine, United States of America

## Abstract

Alzheimer's disease (AD) is the most common progressive neurodegenerative disease known to humankind. It is characterized by brain atrophy, extracellular amyloid plaques, and intracellular neurofibril tangles. β-amyloid cascade is considered the major causative player in AD. Up until now, the mechanisms underlying the process of Aβ generation and accumulation in the brain have not been well understood. Tyro3 receptor belongs to the TAM receptor subfamily of receptor protein tyrosine kinases (RPTKs). It is specifically expressed in the neurons of the neocortex and hippocampus. In this study, we established a cell model stably expressing APPswe mutants and producing Aβ. We found that overexpression of Tyro3 receptor in the cell model significantly decreased Aβ generation and also down-regulated the expression of β-site amyloid precursor protein cleaving enzyme (BACE1). However, the effects of Tyro3 were inhibited by its natural ligand, Gas6, in a concentration-dependent manner. In order to confirm the role of Tyro3 in the progression of AD development, we generated an AD transgenic mouse model accompanied by Tyro3 knockdown. We observed a significant increase in the number of amyloid plaques in the hippocampus in the mouse model. More plaque-associated clusters of astroglia were also detected. The present study may help researchers determine the role of Tyro3 receptor in the neuropathology of AD.

## Introduction

Alzheimer's disease (AD) is the most common neurodegenerative disease known to humankind and a major form of dementia. It impairs basic cognitive functions, primarily memory [1], [2]. The etiology and pathogenesis of the disease are still not yet well understood. AD is characterized by three age-dependent pathological features. The deposition of amyloid plaques occurs mainly extracellularly. These are also called senile plaques (SP), or neuritic plaques. SPs and neurofibrillary tangles (NFTs) are caused by the intraneuronal hyperphosphorylation of Tau protein and apoptotic neuronal death [3], [4], [5]. These features are most evident in the neocortex and hippocampus. As the main component of neuritic plaques, the amyloid β peptides (Aβ) are considered key molecules in the pathogenesis of AD [6], [7]. Aβ peptides are viewed as the culprit of this disease. They act as the main trigger for a series of processes known as the amyloid cascade [8]. This cascade generally culminates in apoptotic neuronal death. These amyloidogenic peptides are derived from an integral membrane protein, called amyloid precursor protein (APP), which is cleaved by the proteases β- and γ-secretase through a two-step proteolytic process. Although APP amyloidogenic processing produces fragments of different lengths, Aβ40 and Aβ42, have 40 and 42 amino acids, respectively, and they are the two most abundant of Aβ [7], [9]–[11]. Aβ42 aggregates at a much faster rate and at a lower concentration than other fragments. Robust evidence confirming the amyloid cascade hypothesis has been gathered from studies of AD transgenic mice carrying human missense mutant APP and presenilin-1 (PS1) genes, which encode mutant human APP and PS1 proteins that can produce much more Aβ, especially Aβ42 [9], [12]. These mouse models share some aspects of human AD, such as amyloid plaques, neuron and synapse loss, and correlative memory deficits.

The Tyro3 family is a subfamily of receptor tyrosine kinases (RPTKs). It comprises Rse/Tyro3, Axl/UFO, and Mer/Eyk [13]–[17]. These three receptors share a ligand-binding ectodomain, a single membrane-spanning domain, and a cytoplasmic tyrosine kinase domain [18], [19]. The *tyro3* gene is expressed during central nervous system neurogenesis and exhibits distinct and highly regionalized patterns of expression in the adult brain [19]–[21]. In human tissues, especially, the highest concentration of expression of *tyro3* mRNA is observed in the brain. Tyro3 is expressed at high levels in the mouse cerebral cortex and hippocampus. Moreover, the highest levels of Tyro3 expression in the brain are associated with neurons [14], [21]–[23]. Two related proteins, the growth arrest specific gene product Gas6 and protein S, have been identified as ligands of TAM family receptors [24], [25]. Gas6 functions as a ligand for TAM receptors and can protect cortical neurons from β-amyloid induced apoptosis [26]. It can also attenuate serum-starvation-induced cell death in the hippocampal and gonadotropin-releasing neurons [27], [28]. Gas6 has also exhibited trophic effects on the survival and proliferation of glial cells in both the central and peripheral nervous systems [29], [30]. Recent reports have shown that Tyro3 receptors are closely related to immunodysfunction in the central nervous system [31]–[33]. The region-specific expression of Tyro3 suggests that it may play an important role in the development and biological functions of the central nervous system. Tyro3/Axl/Mer triple knockout brains have exhibited altered histology and increased rates of apoptosis and cellular degeneration [18], [19]. We have also demonstrated that nerve growth factor (NGF) insufficiency is considered major factors in cholinergic neuronal degeneration in the brains of organisms with AD [34]–[36]. NGF induces both Tyro3 and Axl expression in differentiating PC12 cells, and these receptors interact with TrkA, which is a receptor specific to NGF. Activation of Tyro3 by Gas6 protects PC12 cells from death induced by serum starvation and NGF deprivation [37], [38]. All of these aforementioned observations suggest that Tyro3 receptor may has a protective effect against the progression of AD. To date, however, the functions of Tyro3 receptor in pathology of AD remain unclear.

All of the above indicate that Tyro3 receptor may regulate the formation of AD pathology. In the present study, we used human APPswe transgenic models in vitro and in vivo to determine whether Tyro3 affects Aβ production and Aβ deposition. We observed that the APP processing mediated by BACE1 was inhibited by Tyro3 overexpression in vitro, and the number of senile plaques resulting from Aβ deposition was increased by Tyro3 gene knockdown in an AD mouse model.

## Results

### Effects of Tyro3 overexpression on Aβ production from HEK293 cells stably expressing APPswe mutants

**Figure 1:**
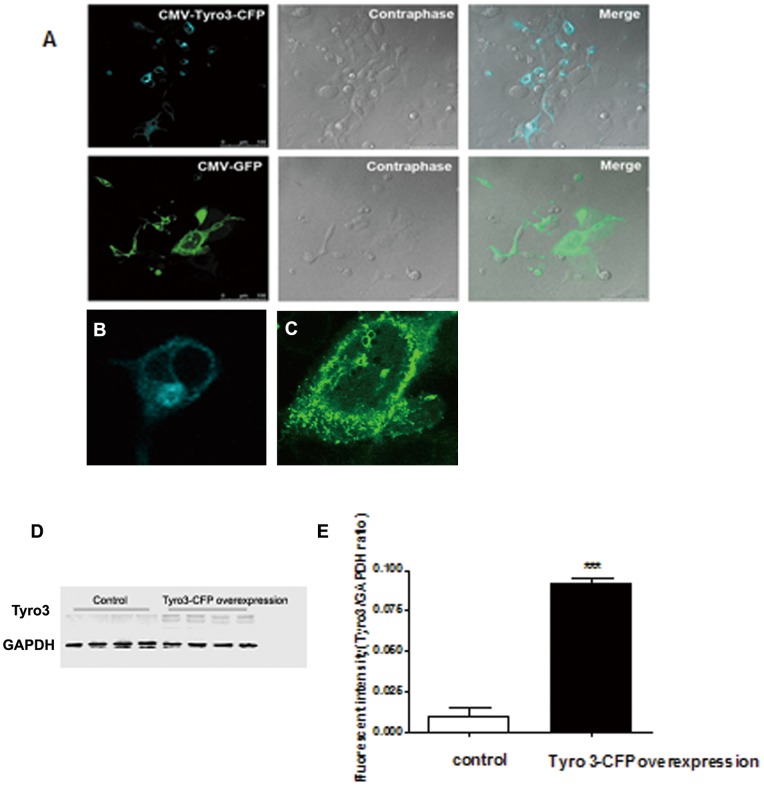
Tyro 3-CFP transiently overexpressing in 293APPswe cells. (A) In live 293APPswe cells, Tyro3-CFP fusion and green fluorescent protein (GFP), phase contrast image, and merged image were observed by confocol microscopy after 24 h of transfection with CMV-Tyro3-CFP and CMV-GFP, respectively. The merged portrait consists of transfected cells, and the bar scale is 100 μm. Transfection efficiency of CMV-Tyro3-CFP in 293APPswe is equal to that of CMV-GFP in these cells. (B) Enlarged image from 293APPswe cells overexpressing Tyro3-CFP. (C) Enlarged image from 293APPswe cells overexpressing GFP. (D) Western blot for Tyro3 of protein extracts from GFP transfected 293APPswe cells and Tyro3-CFP transfected 293APPswe cells. The protein level of Tyro3 in 293APPswe cells transfected with CMV-Tyro3-CFP was found to be higher than in controls transfected with CMV-GFP. GAPDH was used as a loading control. (E) Statistical analysis showed a significant increase in the level of Tyro3 in the Tyro3-CFP overexpressing cells (***P<0.001).

**Figure 2:**
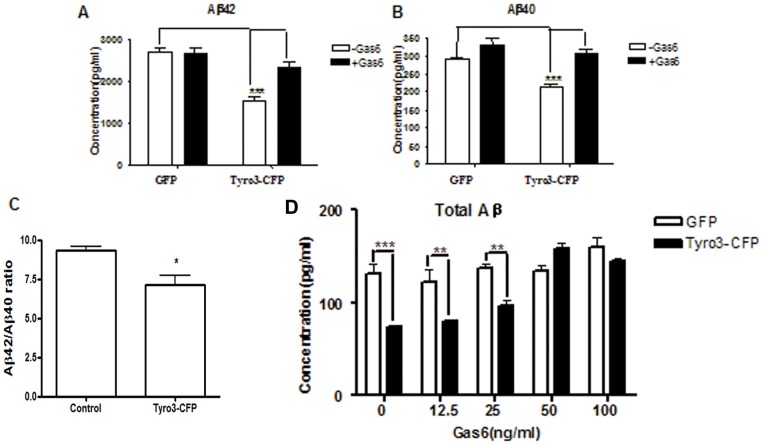
Tyro 3 overexpression decreases the production of Aβ from 293APPswe cells and Gas6 inhibits the effect in a concentration-dependent manner. (A, B) ELISA assay shows that the levels of (A) Aβ42 and (B) Aβ40 are significantly reduced in Tyro 3-CFP transfected cells relative to GFP transfected cells and the effect is inhibited by addition of Gas6 at the final concentration of 100 ng/ml. (C) The ratio Aβ42/Aβ40 is also significantly lower in Tyro 3-CFP transfected cells than in GFP transfected cells. (D) ELISA assay shows that a decrease in Aβ in Tyro-3-transfected 293APPswe cells relative to the GFP transfected controls. Both sets of cells were treated with different concentrations of Gas6 (12.5, 25, 50, 100 ng/ml). Total Aβ was also significantly lower in Tyro-3-transfected cells treated with low concentrations of Gas6 (12.5, 25 ng/ml) than in GFP controls. ***P<0.001, **P<0.01 versus GFP controls (student's t-test or one way ANOVA). Doses of 50 ng/ml or 100 mg/ml caused no obvious changes in the Gas6-treated group.

To determine whether Tyro3 receptor could affect APP processing and Aβ production we first established a cell line, HEK293 cells stably overexpressing APPswe mutants (here called 293APPswe cells). This line is characterized overexpression of human APP695 proteins (110 KD) (Figure S1) and producing excessive Aβ, especially Aβ42 by the APP processing compared with undetectable production of wild type HEK293 cells (data not shown). In the next step, we transfected a Tyro3-CFP chimera transiently to the 293APPswe cells. GFP expression plasmid was used to transfect the cells as a control. After 24 h of transfection, Tyro3 protein overexpression in those cells was clarified using confocal imaging and Western blot analysis (Figure 1). As shown in Figure 1A and B, Tyro3-CFP chimera mainly expressed on the membrane but also in cytosol, in contrast with diffused GFP expression pattern in control cells (Figure 1A and C). Our ELISA analysis showed that Tyro3 overexpression significantly reduced both the production of Aβ42 and Aβ40 (Figure 2 A and B), as well the ratio Aβ42/Aβ40 (Figure 2C). These data suggest that Tyro3 receptor overexpression is related to the generation of Aβ and APP processing.

### Effects of Gas6 on decreases in Aβ production induced by Tyro3 overexpression

Tyro3 can be activated by its natural ligands. To further determine whether the effects of Tyro3 on the production of Aβ depends on its ligand, Gas6, we subjected 293APPswe cells transfected with GFP or Tyro3-CFP to media lacking or containing Gas6 at final concentration of 100 ng/ml. After 24 h of treatment, we observed that Tyro3 overexpression similarly reduced the production of Aβ40 and Aβ42 in the absence of Gas6. However, these effects were completely inhibited in the presence of Gas6 (Figure 2A and B). To exclude the possibility that the inhibitory effect of Gas6 at the final concentration of 100 ng/ml was caused by a high concentration of reagents, we also used 12.5, 25, 50, and 100 ng/ml of Gas6 to treat 293APPswe cells overexpressing Tyro3-CFP and GFP. Tyro3 overexpression was found to significantly reduce the production of Aβ, including Aβ40 and Aβ42, in the absence of Gas6 and in the presence of Gas6 at final concentrations of 12.5 or 25 ng/ml. However, it had no effect on the total Aβ levels secreted by 293APPswe cells in the presence of Gas6 at final concentrations of 50 or 100 ng/ml (Figure 2D). The result also showed that Gas6 inhibits the decreases in Aβ production induced by Tyro3 overexpressing in a concentration-dependent manner. This suggests that the effect of Tyro3 on Aβ production is not only independent of its ligand, Gas6, but can also be inhibited by Gas6.

### Effects of Tyro3 overexpression on BACE1 and CTFs expression

**Figure 3:**
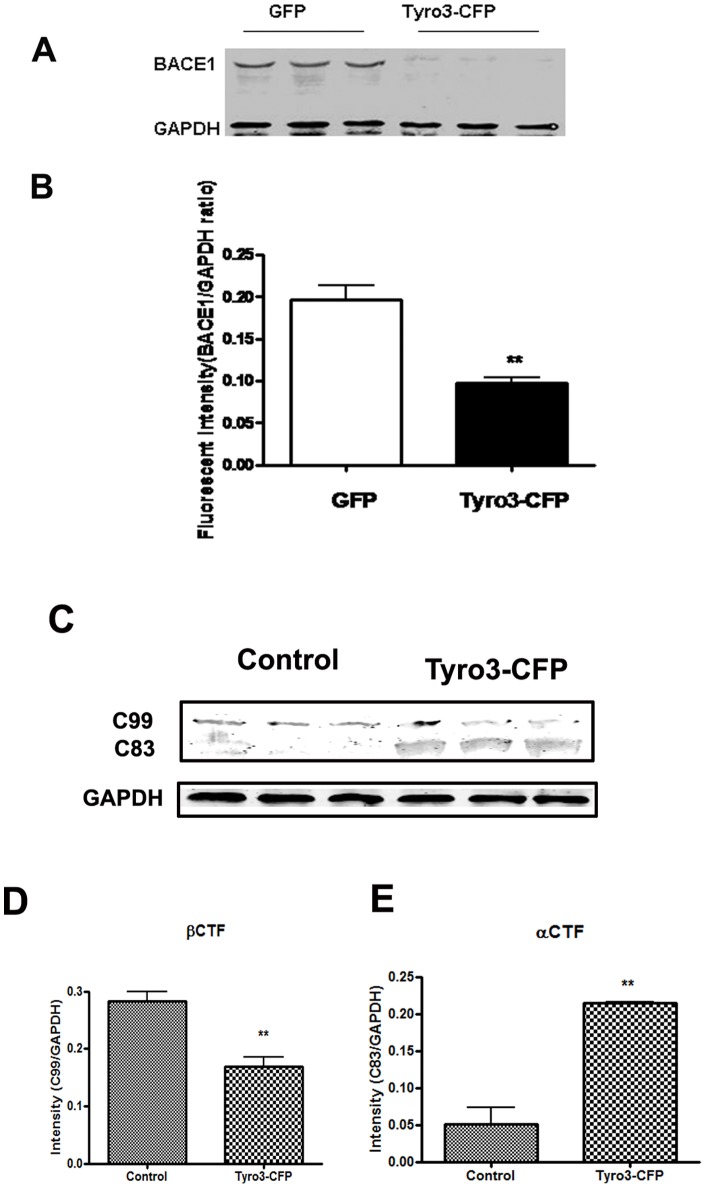
BACE1 protein level and activity are down-regulated by Tyro3 overexpression in 293APPswe cells. (A, B) Western blot analysis of BACE1 showing that the protein levels of BACE1 in extracts from Tyro3-CFP transfected cells have lower fluorescent intensity than GFP controls. (C, D, E) Increase in C99 (βCTF) protein level and decrease in C83 (αCTF) protein level in extracts from Tyro3-CFP transfected cells were detected by western blot analysis compared to that of GFP control. *P<0.05, **P<0.01 versus GFP control (student's t-test).

**Figure 4:**
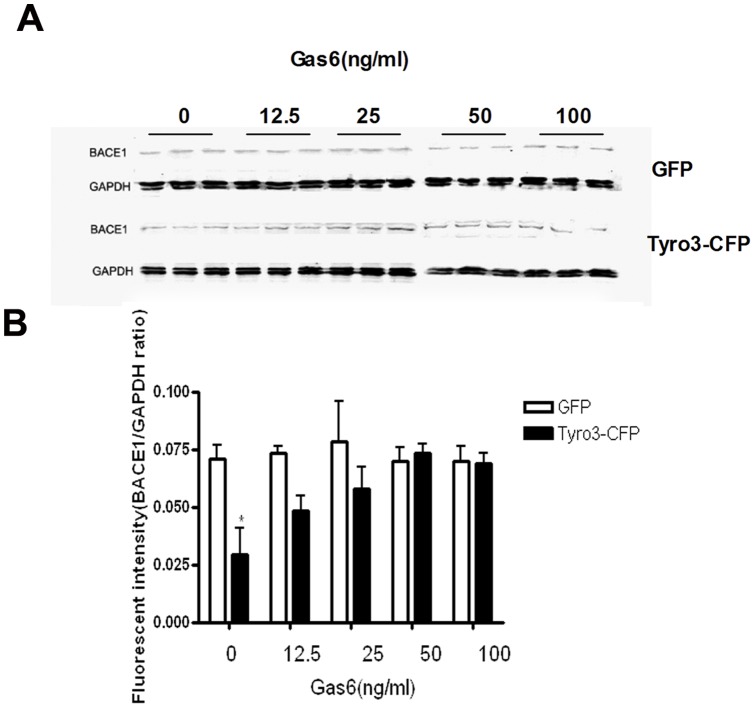
Gas6 inhibits the decrease in BACE1 protein level induced by Tyro3 overexpression in 293APPswe cells. (A, B) Western blot statistical analysis showed that BACE1 levels were significantly lower in Tyro 3-CFP transfected cells than in GFP controls. Gas6 treatment was found to reverse this effect. *P<0.05 versus GFP control (two way ANOVA).

The β-secretase, β-site amyloid precursor protein cleaving enzyme (BACE1), is responsible for initiating Aβ generation. The BACE1 first cuts APP to generate the N terminus of Aβ, producing a membrane-bound C-terminal fragment called C99. Then γ-secretase cleaves C99 to release the mature Aβ peptide. Alternatively, APP can be cleaved by α-secretase within the Aβ domain to generate C83 and preclude Aβ generation [7], [9], [11], [39]. To determine whether the decrease in Aβ production is caused by the alleviation of APP processing induced by BACE1 downregulation, which could be instigated by Tyro3 overexpression in 293APPswe cells, we incubated the 293APPswe cells overexpressing GFP or Tryo3-CFP in the medium in the absence or presence of Gas6 at different concentration of 12.5, 25, 50 or 100 ng/ml. The protein extracts were subjected to Western blot assay and we observed that the protein level of BACE1 is significantly lower in 293APPswe cells overexpressing Tyro3 than in GFP control cells (Figure 3A, B). As the metabolites of APP cleaved by BACE1, C99 (βCTF) also is reduced in Tyro3 overexpressing cells, while, C83 (αCTF) expression is increased by Tryo3 overexpression in 293APPswe cells, simultaneously (Figure 3C, D, E). The result suggests that Tyro3 could not only alter BACE1 protein level but also regulate BACE1 activity. Next, we evaluated the effects of Gas6 on BACE1 protein expression because Gas6 had inhibited the decrease in Aβ production induced by Tyro3 overexpression. There was no statistically significant effect of Tyro3 overexpression was detected in the presence of Gas6 at any final concentration of BACE1 protein expression in the 293APPswe cells compared to control cells, although Tyro3 overexpression in the presence of Gas6 at a final concentration of 12.5 ng/ml tended to reduce the production of Aβ (Figure 4A, B). This suggests that Tyro3 overexpression reduces Aβ production at least in part through downregulation of BACE1 protein expression in 293APPswe cells and that the effects of Tyro3 receptor on amyloidogenic APP processing and Aβ generation are not only independent of Gas6 but can be inhibited by Gas6.

### Effects of Tyro3 receptor partial knockdown on amyloid plaque formation in the CA1 hippocampus and subiculum of 5XFAD transgenic mouse brains

**Figure 5:**
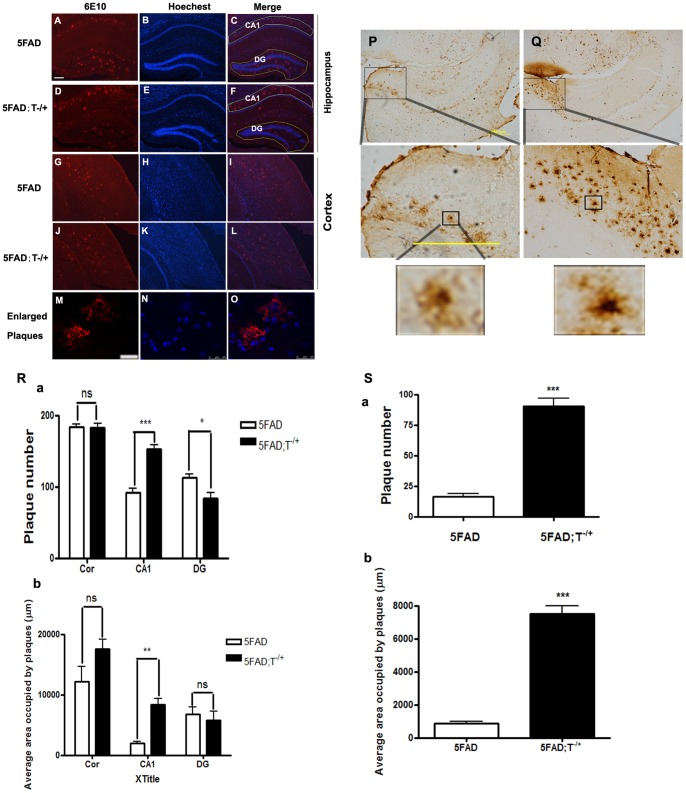
Tyro3 knockdown significantly increases Aβ plaque formation in the CA1 and subiculum area but not in the DG region in 5XFAD mouse brains. A–L), Aβ immunoreactive plaques in the (A–F) hippocampus (G–L) and cortex of (A–C, G–I) 5XFAD transgenic mice and (D–F, J–K) 5XFAD; T−/+ crossed mice, respectively. 6E10 immunoreactive plaques are shown as red fluorescence and nuclei are stained blue by Hoechst. CA1 and DG regions are indicated with lines. Scale bar = 200 μm. (M–O) Enlarged plaques were detected by confocal laser scanning microscopy. Scale bar = 25 μm. (P, Q) Aβ immunohistochemical images showing Aβ-positive plaques in the subiculum of 5XFAD and 5XFAD; T−/+ mice. Scale bar = 500 μm. (R, S) Quantification of plaques showing both the number of plaques (Figure 5R-a, S-a) and average area occupied by plaques (Figure 5R-b, S-b) are increased in CA1 and subiculum areas in 5XFAD; T−/+ mice compared with 5XFAD controls. However, the number of plaques in DG region in 5XFAD; T−/+ mouse brain was much higher than that of 5XFAD controls. ***P<0.001, **P<0.01, *P<0.05 versus 5XFAD transgenic controls (student's t-test or two way ANOVA).

Given that Tyro3 receptor overexpression ameliorated the generation of Aβ, especially that of Aβ42, in APPswe mutant cell model, we anticipated that Tyro3 also would affect Aβ accumulation in the brains of APP/PS1 mutant mice. To determine whether Tyro3 receptor could influence Aβ deposition in vivo, we generated the 5XFAD; Tyro3^−/+^ transgenic mice by crossing 5XFAD mice with Tyro3 gene knockout mice (Tyro3^−/−^). Age-matched 5XFAD transgenic mice were used as controls. As in previous studies, we found that immunostaining with an Aβ-specific monoclonal antibody, anti-6E10, revealed the 5XFAD mice had obvious amyloid deposition in the cerebral cortex and hippocampus at 6 months of age (Figure 5A–C, G–I) [40]. Aβ deposition was dramatically increased in the CA1 region of the hippocampus in 5XFAD; Tyro3^−/+^ mouse brains (Figure 5D–F). This was statistically significantly different from 5XFAD transgenic mice. Although the number of amyloid plaques in the cortex of 5XFAD; Tyro3^−/+^ mice did not exhibit distinguishable differences from 5XFAD transgenic mice (Figure 5G–L, R). Unexpectedly, the number of amyloidal plaques deposited in the dentate gyruses (DG) displayed was slightly lower in 5XFAD; Tyro3^−/+^ transgenic mice than in 5XFAD transgenic mice, as shown in Figure 5A–F, R. However, Aβ deposition showed a much more diffuse pattern in 5XFAD; Tyro3^−/+^ transgenic brains than in 5XFAD controls. The enlarged confocal laser scanning images (Figure 5M–O) verified that the plaques formed extracellularly were similar to those previously described and specific to Aβ [3], [4], [6]. This was confirmed by the staining wild-type mouse brains, which showed almost no immuno-positive plaques for anti-6E10 antibody staining (data not shown). It is noteworthy that the amyloid deposits in subiculum of 5XFAD; Tyro3^−/+^ transgenic brain were vastly more numerous than those observed in the 5XFAD transgenic brain (Figure 5P, Q, S). Moreover, as shown in Figures 5P and 5Q, enlarged images of Aβ-immunoreactive plaques demonstrated that amyloid deposits in 5XFAD; Tyro3^−/+^ transgenic mice were typically smaller but had greater plaque seed density than that of 5XFAD transgenic mice, possibly because of the highly elevated Aβ42 levels in 5XFAD; Tyro3^−/+^ transgenic mice.

**Figure 6:**
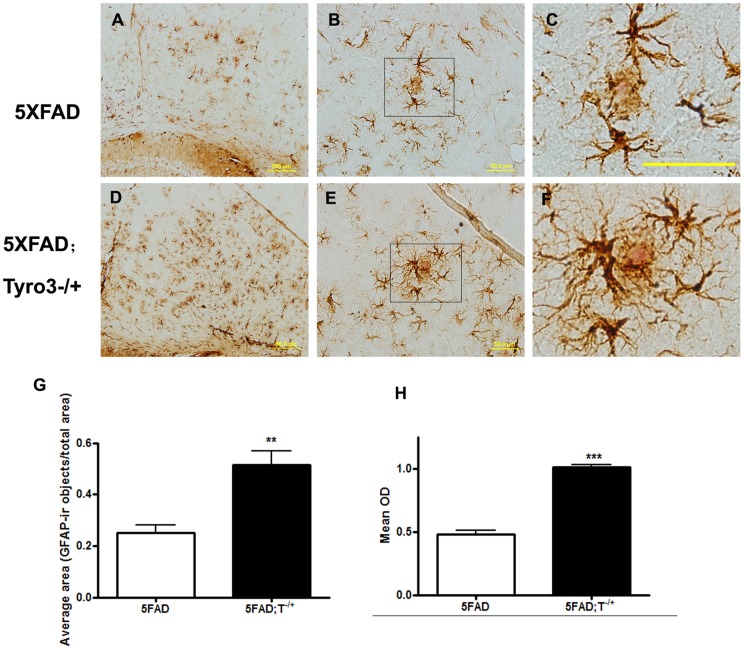
Tyro3 knockdown increases plaque-associated clusters of astroglia in 5XFAD mouse brains. (A, D) GFAP immunoreactive astrocytes (brown) and plaques stained with Congo red dye (dense pink core) in the cortexes of (A) 5XFAD transgenic mice and (D) 5XFAD; T−/+ crossed mice, respectively (Scale bar = 200μm). (C, F) Enlarged images are chosen from a field, which are surrounded by black frames as shown in (B) and (E), centered on a putative amyloid plaque. Scale bar = 50 μm. (G) Area occupied by GFAP-ir cells per total area under observation is increased in brain slices of 5XFAD; T−/+ mice compared with 5XFAD controls. (H) Quantification analysis showing the average optical density (OD) of GFAP-ir occupied area in 5XFAD; T−/+ mice is significantly higher than that of 5XFAD controls. ***P<0.001, **P<0.01 versus 5XFAD transgenic controls (student's t-test).

**Figure 7:**
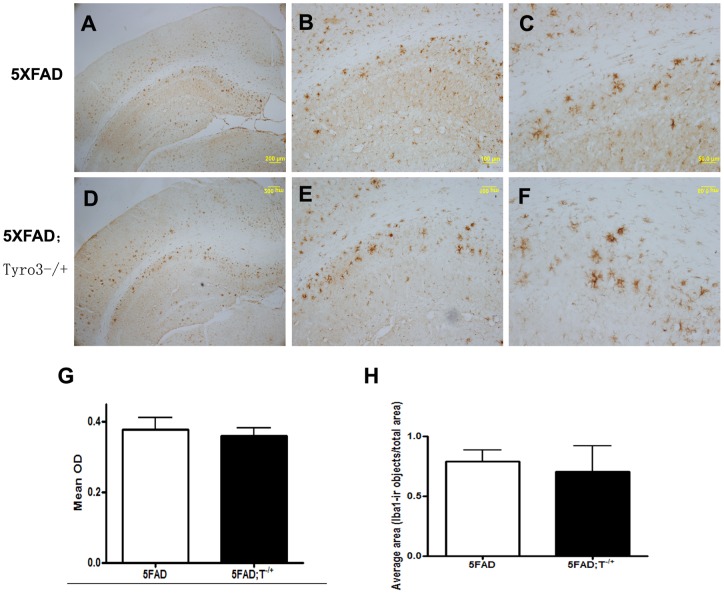
Tyro3 knockdown has no effect on microglial activation in 5XFAD mouse brains. (A, B, C) Iba1 immunostaining in 5XFAD mouse brain slices (Scale bar = 200, 100, 50 μm, respectively). (D, E, F) Iba1 immunoreactive microglia in 5XFAD; T−/+ mouse brain slices (Scale bar = 200, 100, 50 μm, respectively). (G) Quantification of mean optical density (OD) of Iba1 staining showing no significant difference between 5XFAD controls and 5XFAD; T−/+ mouse brains. (H) Statistical analysis of ratio of area occupied by Iba1-ir cells to total area of image showing no significant change between 5XFAD controls and 5XFAD; T−/+ mouse brains (student's t-test).

We also used Congo red dye to stain plaques and detected astrogliosis, simultaneously using astrocyte specific marker GFAP immunostaining. We found that 5XFAD mice had relatively diffuse distribution of GFAP-immunoreactive (GFAP-ir) astroglial cells and congophilic plaque-associated clusters of astroglia (Figure 6A–C). Partial Tyro3 knockdown (Figure 6D–F) caused an increase in the area occupied by GFAP-ir cells and optical density of GFAP stainning around congophilic plaques (Figure 6G, H). To determine whether partial Tyro3 knockdown affects the microglial activation in the 5XFAD mouse brain, we next examined the microglial reactivity in mouse brains using Iba1 (ionized calcium binding adaptor molecule 1) that is specifically expressed in macrophages/microglia antibody. However, no significant difference in the immunoreactivity of Iba1 positive cells was found between 5XFAD transgenic mice and 5XFAD; Tyro3^−/+^ mice (Figure 7).

## Discussion

Although increasing numbers of genetic and pathological studies implicate alterations in APP processing as being central to AD mechanisms, the causative factors influencing Aβ generation and deposition remain unclear. APP has a single membrane-spanning domain, with a long extracellular N-terminal and short intracellular C-terminal region. Aβ is derived from APP, which is cleaved by β-secretase (also called BACE, β-site APP cleaving enzyme) and γ-secretase, sequentially. Normally, the generation of Aβ peptides is at equilibrium and less pronounced than the products cleaved by α-secretase from APP [7], [9], [39]. However, Aβ production was found to be increased significantly by the APP mutations associated with early-onset familial AD [12], [41]. The present study demonstrated that overexpression of APP with the Swedish mutant promotes the production of Aβs, especially Aβ42, in HEK293 cells, as reported previously [42], [43]. In this way, the cell model overexpressing the Swedish mutant APP should be a better tool to research the procedure of amyloidogenic APP processing and Aβ generation associated with AD pathogenesis. In the first step of our study, we explored whether Tyro3 receptor could affect APP processing and Aβ production (because the Tyro3 receptor is preferentially expressed in neurons of the cerebral cortex and hippocampus) and which parts of the brain are involved in AD progression [13], [14], [21], [23]. We found that Tyro3-CFP is expressed mainly on the membrane but also in the cytoplasm. It is very important for Tyro3 receptor to exhibit its normal functions as a receptor tyrosine kinase [13], [14], [18], [19]. We also found that Tyro3 overexpression alleviated Aβ generation in 293APPswe cells. It has been discovered that the natural ligands of Tyro3 receptor are protein S and Gas6. Gas6 is expressed in the brain and has been shown to posses neuroprotective and neurotrophic functions [13], [14], [21], [23], [26]–[28], [37]. Here, we used Gas6 to stimulate Tyro3 receptors to determine whether their effect on APP processing and Aβ production is dependent on their ligand, Gas6. In striking contrast with our expectance, the effect of Tyro3 overexpression on Aβ production in 293APPswe cells was found to be inhibited by the presence of Gas6 in a concentration-dependent manner. Taking into account the presence of serum in the culture medium of the 293APPswe cells, some factors, especially Protein S, which is the other natural ligand of Tyro3 receptors, might stimulate Tyro3 receptor tyrosine kinase [25]. The other possibility is that Tyro3 itself might interact with APP or some other factor related to APP processing. This requires additional analysis.

We next explored how Tyro3 overexpression reduced Aβ generation in HEK293 cells through APPswe mutant proteins. Increases in Aβ production did not seem likely to be induced by up-regulation of APPswe mutant protein expression because we did not observe any changes in APP transgene expression when Tyro3 was overexpressed in 293APPswe cells (data not shown). The amyloidogenic APP processing complex, BACE1, is a predominant β-secretase enzyme responsible for Aβ generation in the brain [39], [44]. Recent studies have shown that Aβ generation, amyloid pathology, electrophysiological dysfunction, and cognitive deficits become abrogated when BACE1^−/−^ mice are bred with APP transgenic mice [45], [46]. We also examined the expression of BACE1 in 293APPswe cells and demonstrated that Tyro3 overexpression could decrease BACE1 protein levels in those cells. This indicates that the decreased BACE1 levels are involved in the mechanism by which Tyro3 receptors affect amyloidogenic APP processing and Aβ generation, as mentioned above. Gas6 treatment also inhibited the effects of Tyro3 receptor on BACE1 expression. Our results suggest that Tyro3 receptor contributes to the inhibition of Aβ production in transgenic cell models, promoting the familial disease mutations in APP by suppressing BACE1 protein expression. One counterintuitive part of our results is that Gas6 impedes the ability of Tyro3 to inhibit Aβ production, which contradicts previous reports of the neuroprotective and neurotrophic effects of Gas6 on Alzheimer's disease-like disorders [13], [14], [21], [23], [26]–[28], [37]. Analyzing those reports and our observations, we speculate that the effects of Tyro3 on APP processing are independent of Gas6-mediated signaling. The manner by which Tyro3 receptors influence APP processing and Aβ accumulation in the progression of Alzheimer's disease requires further study. Fortunately, the progression of AD has been studied in many kinds of Alzheimer's transgenic mice. We used APP/PS1 doubly transgenic mice co-expressing human PS1 and APP transgenes with a total of five FAD mutations including the Swedish mutation in 293APPswe cells [40]. These transgenic mice are characterized by rapid amyloid deposition, which increases with age and reaches massive levels when the mouse is 6 months old. Interestingly, young 5XFAD mice produced Aβ42 almost exclusively as early as 1.5 months of age, while the Aβ40 increase typically lagged 2–3 months behind the increase in Aβ42 levels [40]. This phenomenon can be used to evaluate the role of Tyro3 in Aβ production and accumulation in the development of AD in the brain.

Our studies show that partial knockdown of the Tyro3 receptor promotes Aβ deposition in AD transgenic brains, especially in CA1 region of the hippocampus. The CA1 area is the exact brain region that expresses Tyro3 receptor [23]. It supports long-term potentiation (LTP), an electrophysiological measure related to learning and memory [47], [48]. It is the most important brain region that is impaired in the progression of AD. Normal levels of Tyro3 expression might play a beneficial role against Aβ production, deposition, and toxicity. The present knockdown experiment also suggests the involvement of Tyro3 receptor in reducing amyloid plaques in progression of AD. On the other hand, the present study also provides a clue suggesting that the pathological features of AD are closely associated with hippocampal development: Tyro3 expression in CA1 pyramidal neurons is first detected in 14.5 day old mouse embryos, which is when hippocampal development begins [14], [23]. Notably, the number of amyloid plaques in the subiculum was noticeably higher in 5XFAD; Tyro3^−/+^ transgenic brains than in 5XFAD mice brains. The subiculum is also an important part of hippocampal formation, that it is involved in learning and memory, and that it is among the first parts of the brain to be damaged during AD [40], [48]. Taking this into account, our results suggest that Tyro3 is closely related to hippocampal functions, such as LTP, and so is associated with the pathogenesis of AD and might act as a downstream molecule of amyloidogenic APP processing and the amyloid cascade in the progression of AD, but this could be a factor in turn blocking that process. Although the number of amyloid plaques did not change in cortex – they even decreased slightly in the dentate gyrus – we still found more and smaller sporadic plaques around dentate gyrus field and in the cortexes of 5XFAD; Tyro3^−/+^ brains than in those of 5XFAD mice.

Although we have not quantified Aβ levels in vivo, we speculate that increasing levels of Aβ, especially Aβ42, in the brains of 5XFAD; Tyro3^−/+^ transgenic mice may be responsible for the increased number of amyloid plaques. This is indicated by the fact that the amyloid plaques found in 5XFAD; Tyro3^−/+^ transgenic mice were typically smaller but had more concentrated cores than those found in 5XFAD transgenic mice. The pronounced astrogliosis surrounding the congophilic plaques supports this conclusion because the amyloid pools caused by Aβ accumulation are more attractive to astrocytes [49], [50]. Evidences showed that Gas6 stimulated phagocytic response and regulated activation of microglia, suggesting its receptors playing a role in mediating an anti-inflammatory effect with the simultaneous stimulation of phagocytosis [51]. Here, we performed microglia immunostaining using anti-Iba1 antidody to detect microglial reactivity in 5XFAD brain and 5XFAD; Tyro3^−/+^ mouse brain. As the result, no significant difference was seen in microglial immunoreactivity between 5XFAD brain and 5XFAD; Tyro3^−/+^ mouse brain. The data indicated that the effect of Tyro3 on Aβ accumulation might be independent of Gas6-regulated inflammatory response. It has been shown that the effect of Gas6 on phagocytosis and inflammatory response is associated with activation of Mer and Axl which are the other receptors for Gas6 [52], [53]. In summary, the present data may help researchers discover the role of the Tyro3 in AD neuropathology. However, the exact mechanisms underlying the influence of Tyro3 on APP processing and Aβ generation require further study.

## Materials and Methods

### Ethics Statement

The experimental procedures were carried out in accordance with the Chinese regulations involving animal protection and approved by the animal ethics committee of the China Capital Medical University.

### Cell culture, treatment, and transfection experiments

Human embryonic kidney 293 (HEK293) cell line was purchased from ATCC (Manassas, VA) and the cells stably transfected with human APP695 containing the Swedish APP670/671 mutation (APPswe) expression plasmid (generously provided by Dr. Yizheng Wang) were made using Lipofectamine 2000 (Invitrogen) following the manufacturer's protocol and selected using G418 (Merck) resistance. The cells were maintained in Dulbecco's minimum essential medium (Gibco) supplemented with 10% heat-inactivated fetal bovine serum (HyClone) at 37°C in a humidified incubator containing 5% CO_2_ and G418 at a final concentration of 200 μg/ml. For overexpression experiments, HEK293 cells stably expressing APPswe mutant (are called 293APPswe cells) were transiently transfected with full-length Tyro3-eCFP fusion or eGFP expression plasmids using Lipofectamine 2000 reagent. For Gas6 treatment, cells were treated with Gas6 at different final concentration for 24 h for transfection of Tyro3-eCFP or eGFP. The supernatants were collected and subjected to ELISA assay, and the cells were harvested for Western blot analysis.

Plasmids: pCMV-Tyro3-eCFP construct was generated by insertion of PCR amplified mouse Tyro3 coding region into pCMV-eCFP. pCMV-eGFP expression plasmid was used as control. All these plasmids were generously provided by Dr. Qingxian Lu.

### Western blotting

Culture cell lysates were prepared from experimental cells with the Cellytic buffer containing protease inhibitor cocktail (1∶100, SigmaAldrich) for 30 min at room temperature followed by sonication on ice for 1 min. They were then centrifuged at 12,000 r.p.m. for 30 min at 4°C. The supernatants were collected and total protein levels were measured using a UV 1700 PharmaSpec ultraviolet spectrophotometer by Bradford method according to the manufacturer's procedure (Bio-Rad). Proteins (50 μg) were analyzed on 10% SDS-PAGE gels, and electrophoretically transferred to nitrocellulose membranes (Milipore). Western blot membranes were blocked with 5% nonfat dry milk in 1×PBS for 2 h at room temperature, and further incubated overnight at 4°C with corresponding primary antibodies. Antibodies used for Western blot analysis include mouse anti-APP695 (1∶1000, 13-0200; ZYMED Laboratories), rabbit anti-Tyro3 (1∶500, sc20742; Santa Cruz), rabbit anti-BACE1 (1∶1000, PA1-757; Thermo), and mouse anti-GAPDH (1∶20000, G8795; SigmaAldrich). The primary antibody labeled membranes were then treated with IRDye^TM^ 800 (green) or IRDye^TM^ 700 (red) conjugated affinity purified anti-rabbit or anti-mouse IgG (Rockland) for 1 h. They were then washed three times with PBS containing 0.1% Tween and twice with PBS alone. The positive Western bands were visualized by LI-COR Odyssey infrared double-fluorescence imaging system (American Company LI-COR).

### Sandwich ELISA

The supernatant of cell cultures were collected and placed on ice and protease inhibitor cocktail was added at a 1∶100 dilution and then centrifuged at 12,000 r.p.m. for 30 min at 4°C. The supernatants were loaded on to 96-well plates and soluble Aβ was detected using a total human Aβ ELISA kit (EH025–48; Excell), human Aβ42 ELISA kit (KHB3441; Invitrogen) or human Aβ40 ELISA kit (KHB3481; Invitrogen) in accordance with the manufacturer's instructions. The absorbance was read at 450 nm using a 96 well plate reader.

### Immunohistochemistry with fluorescent and confocal laser scanning microscopy

To render the transfection assay more efficient, live 293APPswe cells transfected with Tyro3-CFP expression plasmid spreading poly-L-lysine-coated coverslips for 24 h were placed on slides with a drop of 75% glycerol in PBS and subjected to observation with confocal laser scanning microscopy (Leica). Six-month-old female mice were deeply anaesthetized with sodium pentobarbital (50 mg/kg, i.p.) and then perfused transcardially with 0.9% sodium solution, followed by paraformaldehyde in 0.1 M PBS. Their brains were immediately removed and transferred to fresh 4% paraformaldehyde overnight at 4°C. The brains were then cryoprotected by infiltration in 30% sucrose in 0.1 M PBS at 4°C until they sank to the bottoms of 50 ml centrifuge tubes. The brains were embedded in OCT compound. Then cryostat sections were cut and allowed to air dry on SuperPlus glass slides. Serial 10 μm coronal sections were prepared and the standard ABC method was used to determine the distribution of amyloid plaques in 5×FAD and 5×FAD; Tyro3^−/+^ mice brains. The cryostat sections were washed with 0.1 M PBS and treated in 0.1 M PBS buffer containing 3% hydrogen peroxide (H_2_O_2_) for 20 min followed by incubation in normal goat serum (1∶20) for 30 min. Then the sections were incubated overnight with mouse anti-Aβ 6E10 (1∶1000, SIG-39300; Covance) or mouse anti-GFAP (1∶500, MAB360; Millipore) or rabbit anti-Iba1(1∶200, wako, Japan) at 4°C in a humidified chamber. After rinsing in the next day, sections were incubated with biotinylated goat anti-mouse IgG (1∶200) for 2 h at room temperature, followed by amplification with streptavidin peroxidase for 1 h. The sections were rinsed and then treated with diaminobenzidine (DAB) diluted in chromogenic substrate for 5 min until positive brown DAB images showed up. For congophilic staining, the immunostained sections mentioned above were incubated in 0.2% Congo red for 5 min and differentiated using alkaline alcoholic solution. They were then rinsed 3 times. The stained sections were dehydrated, cleared, and covered with neutral balsam. Sections were examined and images were collected using a light microscope equipped with a digital camera (Olympus).

For fluorescence immunohistochemistry, sections were permeabilized with 0.2% TritonX-100 in PBS, pH 7.4 for 20 min at room temperature, blocked in PBS buffer containing 5% normal goat serum for 30 min and incubated in the primary antibodies overnight in a humidified chamber at 4°C. After washing with 1×PBS for 3 times for 5 min each. The sections were incubated for 30 min at 4°C and then 30 min at 37°C with Rhodamine (TRITC)-conjugated goat anti-mouse IgG. After washing, the sections were sealed with coverslips with fluorescent protection mounting media (Vector Laboratories). These sections were observed with fluorescent or laser scanning confocal microscopy (Leica).

The number of Aβ-positive plaques in the cortex and hippocampus formation was calculated using Image Pro Plus 6.0 software. Briefly, 20 consecutive sections of each mouse cortex and hippocampus were imaged together and the areas and the total counts of Aβ-positive plaques in sections per six mouse brains of each group were determined using the software. The level of GFAP or Iba1 immunoreactivity was measured by using mean optical density of DAB staining. In addition, ratio of area of object to total area of image was also obtained by the software. The white balance of all images was standardized to eliminate color bias.

### Animals

We used APP/PS1 doubly transgenic mice co-expressing and co-inheriting both human APP and PS1 transgenes with a total of five FAD mutations under transcriptional control of the neuron-specific mouse Thy-1 promoter (5XFAD mice, Tg6799 line) [40]. The 5XFAD mice carry an APP transgene with triple FAD mutations (Swedish mutation: K670N, M671L, Florida mutation: I716V; London mutation: V717I) and a PS1 transgene carrying double FAD mutations (M146L and L286V). The 5XFAD mice were originally purchased from Jackson Laboratory and maintained by crossing heterozygous transgenic mice with C57BL/6J wild type breeders. Genotyping was performed by PCR analysis of tail DNA. Hemizygous 5XFAD mice were crossbred with Tyro3^−/−^ mice and the resultant F1 progeny, including 5XFAD; Tyro3^−/+^ were obtained. The simultaneously obtained offspring of 5XFAD transgenic mice crossed with their littermates were used as controls. Tyro3^−/−^ mice (provided by Dr. Qingxian Lu) with SlKSw/129 background had been backcrossed with C57BL/6J mice for at least 20 generations. All these mice were kept in cages in a controlled environment (22–25°C, 50% humidity, 12 h light/dark cycle). The mice were fed a standard diet and distilled water was available ad libitum.

### Data analysis

All experiments were performed in at least three independent assays. Data are summarized as means ± standard errors. Statistical analysis of the results was performed by Student's t testing for unpaired samples or two-way ANOVA model using Prism4.0 software (GraphPad Software, San Diego, CA, U.S.). *P* values of <0.05 were considered statistically significant.

## Supporting Information

Figure S1 Establishment of HEK293 cells stably overexpressing APPswe mutants. (A) Western blot for APP of protein extracts of wild-type HEK293 cells (293WT) and HEK293 cells overexpressing APPswe mutants (293APPswe). GAPDH was used as a loading control. The level of APP expressed in 293APPswe cells is increased. (B) Relative fluorescent intensity of Western blots shows a significant increase in the levels of APP in 293APPswe cells. Statistical analysis was performed using the student's t-test (**P<0.01).
(TIF)
