# Supplementary figures and images for: Involvement of Receptor Tyrosine Kinase Tyro3 in Amyloidogenic APP Processing and β-Amyloid Deposition in Alzheimer's Disease Models
Source: PLoS One. 2012 Jun 11;7(6):e39035. doi: 10.1371/journal.pone.0039035 (PMC3372537; doi:10.1371/journal.pone.0039035)

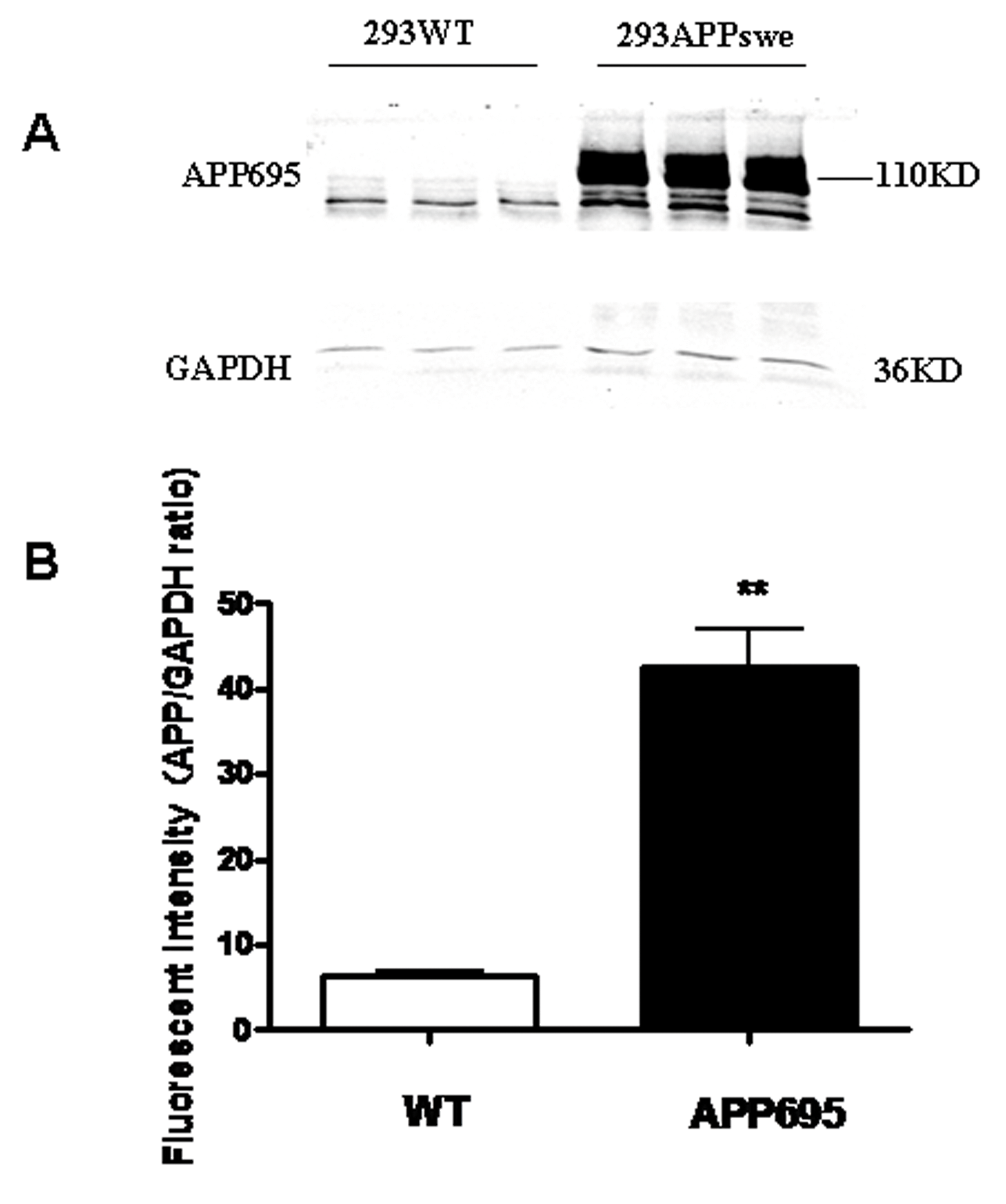

Supplement: Figure S1 — Establishment of HEK293 cells stably overexpressing APPswe mutants. (A) Western blot for APP of protein extracts of wild-type HEK293 cells (293WT) and HEK293 cells overexpressing APPswe mutants (293APPswe). GAPDH was used as a loading control. The level of APP expressed in 293APPswe cells is increased. (B) Relative fluorescent intensity of Western blots shows a significant increase in the levels of APP in 293APPswe cells. Statistical analysis was performed using the student's t-test (**P<0.01). (TIF) [file pone.0039035.s001.tif]
